# Brain tumor-related epilepsy: an overview on neuropsychological, behavioral, and quality of life issues and assessment methodology

**DOI:** 10.3389/fneur.2024.1480900

**Published:** 2024-12-11

**Authors:** Marta Maschio, Fabio Perversi, Andrea Maialetti

**Affiliations:** ^1^Center for Tumor-Related Epilepsy, UOSD Neuro-oncology, IRCCS Regina Elena National Cancer Institute, Rome, Italy; ^2^Freelance Medical Writer, Pavia, Italy

**Keywords:** brain tumor-related epilepsy, anti-seizure medication (ASM), antiepileptic drug (AED), epilepsy, brain tumor, quality of life, neuropsycholgocial tests, behavioral assessment

## Abstract

Brain tumor-related epilepsy (BTRE) is a rare disease in which brain tumor (BT) and epilepsy overlap simultaneously and can have a negative impact on a patient’s neuropsychological, behavioral, and quality of life (QoL) spheres. In this review we (a) addressed the main neuropsychological, behavioral, and QoL issues that may occur in BTRE patients, (b) described how BT, BTRE, and their respective treatments can impact these domains, and (c) identified tools and standardized evaluation methodologies specific for BTRE patients. Neuropsychological disorders and behavioral issues can be direct consequences of BTRE and all related treatments, such as surgery, anti-cancer and anti-seizure medication, corticosteroids, etc., which can alter the structure of specific brain areas and networks, and by emotional aspects reactive to BTRE diagnosis, including the possible loss of autonomy, poor prognosis, and fear of death. Unfortunately, it seems there is a lack of uniformity in assessment methodologies, such as the administration of different batteries of neuropsychological tests, different times, frames, and purposes. Further research is needed to establish causality and deepen our understanding of the interplay between all these variables and our intervention in terms of diagnosis, treatment, psychosocial assessment, and their timing. We propose that the care of these patients to rely on the concepts of “BTRE-induced disability” and “biopsychosocial model” of BTRE, to prompt healthcare providers to handle and monitor BTRE-related psychological and social aspects, as to maintain the patient’s best possible QoL.

## 1 Introduction

For Brain tumor-related epilepsy (BTRE) is a rare disease in which two pathologies overlap simultaneously: brain tumor (BT) and epilepsy (1, 2). These illnesses together with their respective treatments can have a negative impact on a patient’s neuropsychological and behavioral sphere with detrimental effects on QoL (2–5). BTRE patients may require medical attention for a variety of unique concerns: epileptic seizures, possible serious collateral effects of antineoplastic and antiepileptic therapies, physical disabilities and/or neurocognitive disturbances correlated to the tumor site (1, 6). From the patient perspective, in addition to the cancer diagnosis, the burden of epilepsy can add distress to his/her coping with the BT. In fact, literature data indicates that in these patients, the presence of epilepsy is considered the most important risk factor for long-term disability (1, 6).

BTs can be primitive (PBTs) or secondary (SBTs) to systemic neoplasms and often have a non-favorable prognosis (7–9). PBTs represent 1–2% of all cancer cases in adults and their incidence is esteemed in 7.19 cases for 100,000 inhabitants (3); SBTs stem from systemic tumors and can occur in 10–40% of cancer patients (10–12). Neurological disturbances at disease onset or during its course are common both in PBT and SBT, among which neuropsychological and emotional or behavioral alterations (3). PBT can lead to neuropsychological impairment in 30–90% (7, 13–15) of cases, while behavioral alterations are reported in 38–48% of patients (16). SBT-induced neuropsychological and behavioral deficits are reported in up to two-thirds of SBT patients (10–12). In both cases, these disturbances are possibly caused by BT (tumor localization, tumor extension, histological type, molecular index, and tumor degree of growth) and/or its specific treatments (surgery, systemic and supportive therapies) and can induce severe limitations to patients’ functional autonomy (3, 9, 14, 16–18). Regarding all symptoms related to BTs, BTRE is the most common (8–20) and has an incidence varying from 30–70% in PBT patients and almost 20% in patients with SBT (21–29). BTRE constitutes 6–10% of all cases of epilepsy as a whole, and 12% of all acquired epilepsy (22, 23). BTRE is thus a straight consequence of BT (primary or secondary), but the mechanisms underlying epileptogenesis can be different: tumor’s mechanical compression, irritation of peritumoral area, imbalance of vascularization and oxygen demand of the tumor, inflammatory processes, and neurotransmitter disbalance (26–30). BTRE and its treatment significantly aggravate the consequences of the oncological disease (31) for many reasons. The first point to consider concerns the impact of epilepsy itself, which can induce neuropsychological deficits (memory, attention, executive functions) through the alteration of brain networks and their interactions with the rest of the brain (32). Secondly, these patients, in addition to undergoing treatments for the oncological disease (i.e., systemic treatments and surgery) (6, 33), are required to live with the long-term taking of antiseizure medications (ASMs), which can often cause adverse effects (AEs). Among all possible ASM-induced AEs, cognitive and behavioral are common and can further worsen a patient’s autonomy, already impaired by the tumor and its treatments (34). All these factors, often coupled with the label “epileptic,” can cause the patient to feel extremely frustrated when attempting any type of social and/or interpersonal relationship, significantly worsening their QoL (1, 3, 35). Based on these factors, taking care of these patients must include the new concept of “BTRE-induced disability.” From this brand-new perspective, monitoring a patient’s profile through the means of standardized tools assessing neuropsychological, emotional, and QoL domains can provide important information. Unfortunately, it seems there is a lack of uniformity in methodologies used to evaluate these patients to date, such as administration of various batteries of neuropsychological tests for several purposes and within various timeframes.

Thus, we conducted a literature search to (a) review the main neuropsychological, behavioral, and QoL issues that may occur in BTRE patients; (b) describe how BT, BTRE, and their respective treatments can impact these domains; (c) identify tools and standardized evaluation methodologies specific for BTRE patients.

To provide a comprehensive overview of studies that explore all these topics, we searched the available literature using the PubMed database and Cochrane database, selecting articles from 2000 to 2023. Terms used for the search included the following (but not only) words: “BTRE,” “BT,” “quality of life,” “neuropsychological deficit,” “emotional disturbances,” “behavioral alteration,” “sexual dysfunctions,” “neuropsychological evaluation,” “antiseizure medications,” “side effects,” “chemotherapy,” and “radiotherapy.”

## 2 Neuropsychological issues

### 2.1 Impact of brain tumors

Literature data on the etiopathogenesis of neuropsychological disorders in BTs highlight how the tumor itself is the first cause linked to the appearance of deficits in this patient population (36–39). The neoplastic disease can in fact induce neuropsychological disturbances not only through direct damage to the brain structures affected, but also through the involvement of nervous connections that these structures have with the rest of the brain (3, 36). These changes include direct tissue damage via necrosis, compression of neural structures due to mass effect from tumor and surrounding edema, and infiltrative growth into critical fiber pathways and networks (3, 36).

#### 2.1.1 Location, lateralization, and diffuse networks

Localization and lateralization are associated with a variety of neurological patterns of dysfunction and neuropsychological effects, but literature evidence is somewhat contrasting (40–44).

Several studies have shown that right-sided BT or interventions are associated with a lower risk of cognitive impairment, sometimes regardless of the precise tumor localization within the hemisphere (45–54), and that left-hemisphere interventions consistently induce cognitive decline and are associated with lower performance in terms of global function, language, and attention (55–57). Other studies suggest that laterality did not influence outcomes, while others suggest that tumors in the right hemisphere are associated with worse cognitive outcomes compared to left-sided ones, including worse attention and information processing speed (58, 59), worse improvement in verbal memory over a five-year follow-up period despite less impairments at baseline (48), and persistent visuospatial cognitive deficits in some patients (60). Thus, left-hemisphere tumors seem to cause mainly verbal and memory disorders, while right-hemisphere tumors seem to affect non-verbal domains such as visuospatial learning processes and abstract reasoning abilities (3, 42, 61, 62). Nonetheless, the correlation between lateralization and cognitive impairments is not always linear, cognitive deficits seem to not be selective and domain-specific, and patients rather tend to exhibit a global impairment, even in those cases where deficits were related to tumor localization (38, 41). A recent systematic review on 142 studies on the matter found that, although BT localization is one of the most studied variables, the evidence remains conflicting due to methodological and study population differences. Tumor location and laterality do overall appear to influence cognitive outcomes, but the detection of those effects depends on the administration of appropriate cognitive tests (44).

Beyond traditional localizationism, recent awake surgery and fMRI studies highlighted that it is not a single brain region that controls neuropsychological functions or behavioral and emotional regulation, but rather extensive cortical and subcortical networks, connected with different brain areas, even spatially distant from each other (63). For example, current language models propose functional interactions of distributed temporal, frontal, and parietal brain regions within a prevalently left-lateralized neural network: the left-lateralized dorsal stream for phono-articulatory processes and the bilateral ventral stream for semantic processes (64); but language network seems to also be related to other cognitive functions. A recent case–control study on 65 BT patients showed that damage to the left arcuate fasciculus affected postoperative functional ability through verbal short-term memory, working memory, and global cognition in patients with left eloquent hemisphere lesions (65). Other important networks include the default mode network, comprised by the posterior cingulate cortex, precuneus, lateral parietal area, medial prefrontal cortex, and medial temporal area, and involved in multiple neuropsychological functions including episodic memory, prospection, social cognition, and emotions (66–68); the executive control network formed by fronto-parietal areas and involved in fast and flexible goal-directed behavior; the attention network, composed of two separate pathways: dorsal and ventral, consisting of the gyri adjacent to the intraparietal sulcus and frontal eye field in both hemispheres, which is active during tasks that require voluntary attention (66, 68, 69); and the salience network, which includes the anterior insula and dorsal anterior cingulate cortex, the amygdala, ventral striatum, and substantia nigra/tegmental area, and is involved in a variety of functions including self-awareness, social behavior, integrating sensory information, emotional processing, and cognitive functioning (66, 68, 70). BT-induced damage at any point of these diffuse networks can cause the onset of motor, neuropsychological, or behavioral deficits, notwithstanding the localization of the tumor (63–65). By tailoring individual multimodal therapeutic strategies not only to the nature and course of the lesion, but also to the instability of the disrupted connectome and the constant re-adaptation of the large-scale distributed cortico-subcortical networks, we can improve care of patients suffering from BT, epilepsy, and BTRE (63).

#### 2.1.2 Histological type and tumor degree of growth

Histological type and tumor degree of growth seem to have a relation with the type and severity of cognitive disturbances experienced by patients (43, 44). Studies on patients with BT and BTRE evidenced differences in their cognitive profiles, according to the histological type and therefore the degree of tumor growth (32, 44). It seems that patients with high-grade glioma (HGG) present more complex neuropsychological sequelae and more severe deficits, especially in the language domain, compared to those with low-grade glioma (LGG) (41, 44). Nonetheless, some studies indicate patients with HGG more prone to neurocognitive improvements compared to those with LGG (44, 71, 72); this possibly due the fact (a) HGG-related cognitive deficits might be more dependent on higher incidence of intracranial hypertension, and (b) patients with HGG have had little time for rewiring of brain circuitry; thus, cognitive recovery after HGG resection may be facilitated by removal of the physical tumor, whereas in LGG functional reorganization may have already taken place with some functions being subserved by other brain regions or networks (41, 44, 73).

These points highlight the importance of the timing of neuropsychological assessments, which may yield different and conflicting results in relation to HGG/LGG status and available time to recover after intervention.

#### 2.1.3 Tumor’s molecular profile

Molecular markers have been proposed to affect neurocognitive performance as variations in glioma biology can influence can result in disturbed neuronal communication. One study investigated the relationship between executive function, memory, and psychomotor speed and the intratumoral expression of several markers in untreated patients with diffuse glioma; after correction of tumor volume and location, significant associations were found between psychomotor speed and expression levels of CD3 and IDH-1; memory performance and IDH-1, ATRX, NLGN3, BDNF, CK2Beta, EAAT1, GAT-3, and SRF expression; and executive functioning and IDH-1, P-STAT5b, NLGN3, CK2Beta. Other independent associations include expression of P-STAT5b, CD163, CD3 and Semaphorin-3A after correcting for histopathological grade (74).

Beyond being an important marker for BT survival, IDH status thus seems to be consistently associated with different neurocognitive disturbances. Derks and colleagues showed in a cohort of 54 diffuse glioma patients how patients with isocitrate dehydrogenase-mutated (IDH-mut) glioma have a better prognosis but suffer more often of epilepsy with respect to patients with IDH-wild type glioma, who are generally older and more often have neuropsychological deficits (75). In this study authors explained the incidence of cognitive deficit such as the manifestation of lower alpha band functional connectivity in IDH-wild patients, regardless of age and presence of epilepsy, confirming the results of previous research (75, 76). Another study on 119 patients found that IDH1-wild type show reduced neurocognitive functions compared with those with IDH1-mut malignant gliomas, and that lesion volume is inversely associated with neurocognitive functions for patients with IDH1-wild type, but not IDH1-mut tumors (73). These findings pair well with the hypothesis that patients with IDH1-wild tumors, which are generally more aggressive and grow faster, present with more severe deficits due to greater lesion momentum, which may impede compensatory neuroplasticity and cerebral reorganization in the short-term; on the other hand, the lack of circuitry rewiring might result in better long-term recovery after tumor removal, as discussed above for HGG/LGG gliomas (41, 44, 73).

### 2.2 Impact of brain tumor treatments

#### 2.2.1 Neurosurgery

Regarding the impact of BTs’ treatments on neuropsychological issues, the first that must be considered is neurosurgical intervention. Neuropsychological deficits would appear to be focal and specific, in contrast to those caused by radiotherapy (RT) and chemotherapy (CT), which seem to be diffuse (3, 44).

Modern surgery procedures such as awake surgery with intraoperative mapping and electrical stimulation, and real-time monitoring allow for more precise resection of the tumor without damaging surrounding tissue (14). Compared to tumors operated with general anesthesia, those operated through awake craniotomy are associated with better neuropsychological outcomes at six-month follow-up, especially if located in the parietal and insular lobes (77). Awake surgery with intraoperative mapping for visual, cognitive, and haptic functions was shown to decrease long-term neuropsychological, neurological, and QoL morbidity, and to increase the extent of resection in patients with giant insular gliomas, as well as to help preserve spatial working memory and visuospatial cognition in patients with right frontal gliomas (44, 77, 78). In general, awake surgeries tend to result in more positive effects on neurocognitive domains (79).

Regarding the extent of tumor resection, several studies have shown that it is not associated with cognitive outcomes (44). The possible involvement of the healthy tissue surrounding the peritumoral area and/or any pre- or perioperative complications seem to cause neurological and neuropsychological disturbances (3); however, one study on total vs. supratotal resection of radiologically presumed LGGs found that only praxis (but not memory, language, and fluid intelligence) was better in the total resection group immediately after surgery (although this difference reversed after 3 months); nonetheless, the supratotal resection group experienced better recovery of executive functions, with the difference in praxis reversing after 3 months, and improved seizure control (80).

Literature data on the topic is, again, contrasting. Some studies indicate a post-surgery cognitive deterioration followed by partial/full recovery or even improvements in subsequent months; some reveal no effect or improvement in cognition after tumor resection; others found mixed results, with some patients either improving, declining, or remaining the same; finally, some studies found that the same patient could improve in some cognitive domains and remain the same or decline in others (44). Reasons for this rely on different outcomes tested, types of tests used, differences in tumor’s and patient’s characteristics, type and extent of surgery, chosen timepoints, etc. For more information on the matter, we refer to the systematic reviews by Ng and colleagues (79) and Kirkman and colleagues (44). The former found positive effects of surgery on attention, language, learning, and memory (but still with impairments in a wide range of cognitive functions compared to healthy controls), and negative effects on executive functioning in the immediate postoperative period and at 6-month follow-up (79); on the other hand, the latter found that most studies indicate that BT resection does impair neurocognitive functions postoperatively (44). Regardless of the postoperative period, both these and other studies concord on the fact that most of these deficits are transient and recover over few to several months, and that awake surgeries tend to result in better neuropsychological outcomes (5, 14, 44, 79, 81–83).

#### 2.2.2 Radiotherapy

RT can play a role in the onset of neuropsychological deficits (3, 84). Despite the fact that some studies identified mixed results or no effects on neuropsychological outcomes, RT seems to be one of the factors most strongly associated with adverse cognition in patients with BT, and neuroimaging correlates of the cognitive decline have been identified (44). The different effects of RT depend on the radiation dose, the different neuropsychological investigations undertaken and their timepoints (i.e., length of follow-up), and the specific irradiated anatomical structures and their laterality.

There are several types of RT schemes, in which different parts of the brain can be irradiated. The whole-brain RT implies the irradiation of the entire brain and brainstem (85), with cognitive impairment being reported in 40–50% of long-term brain tumor survivors (86). Recent advances in brain imaging and RT techniques allowed a reduction of normal brain tissue irradiated at high radiation doses, consequently minimizing the incidence of treatment-related AEs (87). These new radiation techniques include conformational RT, stereotaxic RT, and imagine-guided RT. Although many efforts have been made to minimize the appearance of neuropsychological disturbances as treatment-related complications, literature data reports radiation-induced cognitive deficits in 30% or more of patients alive at 4 months after partial or whole brain irradiation. For those living over 6 months, that number may rise to 50% (88–90).

A possible explanation of cognitive impairment is represented by the progressive inflammatory action of RT on damaged tissue and on surrounding healthy regions, which can cause the appearance of neuropsychological and behavioral disorders (3). Sheline and colleagues (91) classify AEs of radiation therapy into three main categories, based on the time to onset of symptoms: acute encephalopathy (2 weeks), early-delayed encephalopathy (1–6 months), and late-delayed encephalopathy (>6 months). In the first two cases, factors as edema in the peritumoral area, inflammation of healthy tissue, and rupture of the blood–brain barrier can contribute to the development of cognitive disorders. These include mainly memory and attention and are usually reversible and resolve spontaneously (84, 85, 91). On the other hand, the late-delayed effect can cause large damage to subcortical white matter (necrosis), demyelination, and vascular abnormalities, with severe, irreversible, and progressive alterations to nervous structures, even more than 6 months post-irradiation. These alterations involve working memory, attention, executive function, cognitive flexibility, and processing speed (84, 87, 91). A retrospective longitudinal study on BT patients treated with RT showed how RT-related atrophy is one of the causes contributing to cognitive decline, and that subcortical structures such as the amygdala, thalamus, putamen, pallidum, and particularly the hippocampus, show a significant degree of atrophy after 1 year and taking an average dose of 30 Gy (84). Studies with short follow-up might thus not be able to identify late-emerging, RT-related cognitive declines (44).

#### 2.2.3 Chemotherapy and other systemic therapies

Regarding CT-induced neuropsychological adverse effects, it should be noted that these can be difficult to distinguish from those potentially caused by other treatments such as surgical resection, RT (3, 14, 92), ASMs AEs, or from disease progression (14). Differently from RT, whose effects can onset even months/years after treatment (3, 14), CT-induced side effects generally appear immediately after or at the end of the scheduled therapeutic cycle (3). The appearance of CT-induced cognitive AEs could be related to the accumulation of high levels of drug in the brain, favored by RT disruption of the blood–brain barrier; neurotoxicity; demyelinating damage; microvascular lesions; decreased mechanisms of neurogenesis; oxidative stress; hormonal processes; and neurochemical milieu alterations (41, 93, 94). Consequently, CT-induced cognitive disorders can be variable and heterogeneous.

Among CT therapy regimens for the treatment of BT, CT-induced neuropsychological AEs in patients treated with temozolomide and RT are not reported (3, 95, 96), while vincristine, carmustine, and lomustine treatment can cause toxic reactions with the appearance of dose-dependent neuropsychological disorders affecting memory, attention, and executive functions (14, 97). The onset of late cognitive deficits has also been reported in glioma patients, years after radiation and procarbazine, lomustine, and vincristine CT (14, 98). No neuropsychological AEs are reported for inhibitors of vascular endothelial growth factor such as bevacizumab in patients with recurrent HGG (3, 99–101). Recently, new experimental therapies such as immunotherapy and targeted therapies have been introduced for the treatment of central nervous system tumors, including BT. However, no reports on possible treatment-induced neuropsychological deficits are available (7, 102–105).

Further neuropsychological side effects can be caused by supportive or steroid therapy, generally used for the treatment of cerebral edema corticosteroid therapy can induce dose-dependent AEs on neuropsychological functions such as global cognitive deterioration, although this happens in rare cases (3, 7, 40, 106).

### 2.3 Impact of brain tumor-related epilepsy and antiseizure medications

BTRE can induce or worsen the neuropsychological deficits induced by BT and its treatments (1, 2, 8, 34). Seizures alone can have negative effects on cognitive functions not during the critical episode itself, but also due to a post-ictal state that usually implies a period of significantly decreased cognitive ability (3). There is also increasing evidence that interictal abnormalities can result in cognitive impairment, although shorter than that in the post-ictal period (3). Epileptiform abnormalities, inter-ictal spike, and/or spike–wave patterns represent in fact transient events that can temporarily alternate neural mechanisms, resulting in a transitory cognitive impairment (3, 107, 108). Epilepsy can cause neuropsychological deficit due to the alteration of brain networks that support different cognitive functions, such as memory, language, praxis, executive functions, and social cognition (109). This impairment can differ according to the site of epileptic focus, the duration, and the type of epilepsy (32).

Regarding ASM therapy, studies on patients with non-oncological epilepsy and showed that the use of ASMs can induce AEs which can affect various functional domains: physical, neuropsychological, and behavioral; however, these AEs are more frequent in BTRE patients than in the rest of the population with epilepsy (1). For this reason, there is a need to separate the side effects of ASMs well from other tumor- and treatment-related comorbidities. This requires careful medical history collection, in which it is fundamental to establish the timing of symptom onset in relation to the introduction of an ASM or a change in ASM dose (110).

Old-generation and/or enzyme-inducing ASMs showed a higher incidence of AEs and possible interactions with systemic therapies compared to newer generation and/or non-enzyme-inducing ASMs (20–40%) (1, 8, 21) (Table 1). To date, studies exploring the effect of older, enzyme-inducing ASMs on neuropsychological functions are few and they mainly involve non-oncological patients with epilepsy; one study comparing patients with BTRE and healthy controls found that the presence of glioma was associated with significant reductions in information processing speed, psychomotor function, attentional functioning, verbal and working memory, executive functioning, and health-related QoL; the burden of epilepsy was associated with significant reductions in all cognitive domains (except for attentional and memory functioning) and was primarily related to the use of ASMs; and the decline in QoL was mainly attributed to lack of complete seizure control (34). Some studies evaluating the effects of newer, non-enzyme-inducing ASMs on neuropsychological functions in patients with BTRE are available (Table 1); nonetheless, as for older ASMs, studies are few, often monocentric, and/or involve small samples compared to studies on patients with epilepsy only. Overall, the most important neuropsychological AEs related to new ASMs in this particular patients’ population are not different between those observed in those with epilepsy without BT, but they tend to occur more often, as well as being affected by other therapies and by the neurocognitive deficits related to the presence of the BT and its features (110–113).

**Table 1:** Neuropsychological and behavioral adverse events of ASMs in patients with BTRE.

ASMs	Adverse effects	Effect on drug metabolism	References
First generation			
Carbamazepine (CBZ)	Cognitive slowing; sedation	Enzyme inducer	(111, 259)
Clobazam (CLZ)	No evidenced in BTRE	Non-enzyme inducer	(185, 259)
Phenytoin (PHT)	Cognitive slowing; coordination disturbances; insomnia, depression.	Enzyme inducer	(112, 259)
Phenobarbital (PB)	Cognitive slowing.	Enzyme inducer	(34, 111, 259)
Primidone	No evidenced in BTRE	Enzyme inducer	(259)
Valproic acid (VPA)	Psycho-motor and cognitive slowness, fatigue, somnolence	Enzyme inhibitor	(111, 144–146, 259)
Second generation			
Gabapentin (GBP)	Drowsiness	Non-enzyme inducer	(149, 259)
Lamotrigine (LTG)	Agitation depression, concentration impairment, memory impairment, encephalopathy, somnolence, hallucination, psychosis, insomnia	Non-enzyme inducer	(148, 259)
Levetiracetam (LEV)	Behavioral changes; aggressiveness; agitation; irritability; anxiety; fatigue; somnolence, dizziness; depression; psychosis.	Non-enzyme inducer	(112, 113, 144, 145, 151–157, 196, 259)
Oxcarbazepine (OXC)	Fatigue; confusion; dizziness	Enzyme mixed inducer/inhibitor	(111, 150, 259)
Pregabalin (PGB)	Dizziness, concentration problems, depression, fatigue, erectile disfunction	Non-enzyme inducer	(151, 181, 182, 259)
Tiagabine (TGB)	Tiredness; no other AEs are reported in BTRE patients’	Non-enzyme inducer	(147, 259)
Topiramate (TPM)	Language and memory disturbances; somnolence, dizziness	Enzyme inducer	(169–171, 259)
Zonisamide (ZNS)	Cognitive alterations: verbal fluency, processing speed; somnolence	Non-enzyme inducer	(183, 184, 259)
Third generation			
Brivaracetam (BRV)	Anxiety; agitation; depression; fatigue; dizziness.	Enzyme inhibitor	(172, 259)
Cenobamate (CBN)	No studies available on BTRE patients.	Enzyme mixed inducer/inhibitor	(259)
Eslicarbazepine (ESL)	Fatigue, somnolence, dizziness	Enzyme mixed inducer/inhibitor	(187, 259)
Lacosamide (LCM)	Dizziness; fatigue; somnolence; instability, memory impairment, irritability	Non-enzyme inducer	(148, 173–180, 259)
Perampanel (PER)	Dizziness; fatigue; somnolence; aggressiveness; agitation; anxiety; irritability.	Non-enzyme inducer	(158–165, 167, 259)

Based on this evidence, the choice of ASMs in patients with BTRE must take into account the drug’s efficacy in controlling seizure, but also consider the possible incidence of ASMs’ AEs on neuropsychological, emotional, and behavioral domains which are already burdened by the tumor and its treatments (1, 34, 110, 111, 113, 114) (Table 2).

**Table 2:** Considerations on the use of different ASMs in patients with BTRE.

ASM	Considerations	References
First line management, monotherapy		
Levetiracetam	Caution in case of frontal tumors or with neuro-psychiatric disturbances as it can prompt agitation, aggressiveness, psychotic episodes, and worsen mood Caution in case of anxiety and anxiety disorder Caution in case of concomitant use of corticosteroids May improve sexual functioning	(113, 151–157, 166, 208)
Lamotrigine	Caution in case of insomnia Psychiatric AEs seem uncommon Could help improve mood Discontinuation could worsen mood May improve sexual functioning	(110, 148, 208)
Oxcarbazepine	Psychiatric AEs seem uncommon Can have a positive effect in the psychiatric sphere (e.g., as mood stabilizer) Discontinuation could worsen mood May improve sexual functioning	(110, 111, 150, 208)
Topiramate	Caution in case of language deficits and/or lesions affecting language brain areas/networks Can cause psychiatric AEs including psychosis, aggressive behavior, and depression May cause sexual disfunction	(110, 169–171, 208)
Zonisamide	Caution in case of language deficits and/or lesions affecting language brain areas/networks Neuropsychiatric AEs seem uncommon It might cause reversible erectile disfunction	(183, 184, 206)
Lacosamide	Could induce dizziness, drowsiness, fatigue, somnolence, and confusion	(148, 174–180)
Perampanel^a^	Caution in patients with neuropsychiatric disturbances as it can prompt agitation, aggressiveness, and fatigue Consider in patients with sleep disturbances / insomnia	(158–165, 167)
Second line management, add-on		
Valproic acid	May decrease libido Discontinuation could worsen mood Could mitigate AEs of levetiracetam	(111, 144–146, 208)
Brivaracetam	Caution for possible psychiatric adverse effects	(172)
Pregabalin	To be considered especially in patients with neuropathic pain or anxiety as it may improve anxiety and QoL	(151, 181, 182)

The present table does not account for ASMs’ efficacy and is based solely on type and incidence of adverse events. These considerations may help guide clinicians in the choice of the appropriate ASM according to the drug’s safety profile, which should be taken into account alongside the drug’s efficacy and the patient’s characteristics and preferences. E.g., the use of an ASM affecting verbal fluency might not be the best choice if the tumor is located in Broca’s area. A patient highly valuing his/her sexual life might prefer ASMs which do not cause sexual dysfunction, even if they end up being less effective in controlling seizures.

AEs, adverse events; ASM, antiseizure medication; QoL, Quality of Life.

a The use of this drug as monotherapy or first-line treatment versus as an add-on depends on the type of regulatory approval.

## 3 Emotional and behavioral issues

The etiopathogenesis of emotional and behavioral issues in BTRE patients is rather complex, since the fact they are possibly caused by the association of different factors: damage induced by the two pathologies, possible AEs induced by their respective treatments, and psychological distress related to both diseases. Although identification and treatment of emotional and behavioral issues do not represent the main objective in taking care of BTRE patients, their incidence is quite high (5) with a significant impact on self-perceived QoL and overall survival (115–117).

### 3.1 Impact of brain tumor and related treatments

Among patients with BT and without epilepsy, the prevalence of emotional and behavioral changes seems to occur frequently, with a 38–48% prevalence rate, although they could be often misdiagnosed or undertreated (16). As mentioned above, these disorders are possibly caused by two main factors: organic damage induced by BT and its treatments, and disease-related distress (3, 16). Regarding organic damage, literature data reports that BT and the surrounding edema can cause neuroinflammatory reactions with structural and functional alterations of brain regions and networks critical for the regulation of emotions and behavior (16, 118). Concerning specific brain regions, evidence showed that tumor location, but not extension, is associated with the type of psychiatric disturbance: frontal lesions are linked with depression, apathy, hypermotor and disinhibited presentations, temporal-limbic tumors with panic attacks, and pituitary tumors with depression and anxiety due to hormonal dysregulation of the hypothalamic–pituitary–adrenal axis (16). Tumors located in the thalamus, basal ganglia, and reticular formation are associated with fatigue, lethargy, somnolence, and apathy (16).

However, recent studies highlighted that emotional disorders may be caused by the alteration of a specific subcortical network such as the left striatum and its connections with the rest of the brain (119, 120). Campanella et al. (120) highlight that patients with BT may show behavioral and emotional regulation deficits or personality changes due to the alteration of different neural networks, caused by BT. In a population of 71 BT patients, authors observed that temporal-limbic areas are critical for processing emotions at the perceptual level (e.g., emotion recognition) while frontal lobe regions are involved when a higher level of mentalization or mental abstraction is required (theory of mind, empathy) and this would explain the different behavioral pattern of symptoms exhibited by patients (120).

BT’s treatment-induced emotional and behavioral AEs by organic damage are also reported. Neurosurgical intervention and RT may possibly induce dysregulation of the hypothalamic–pituitary–adrenal axis with mood alterations, depression, and anxiety (16, 121). Potential CT-induced neuropsychiatric AEs for some chemotherapeutic agents such as mood alterations for methotrexate and pemetrexed, depression for vincristine and etoposide, and mania for procarbazine have been described as well (16, 122–124). Although glucocorticoids are currently used as standard therapy for the treatment of tumor-induced brain edema (16, 125, 126), their possible AEs within the behavioral sphere are well documented and can include depression, anxiety, dysphoria, mania, delirium, insomnia, and hyperphagia (127–129).

Finally, another important factor that deserves attention is the disease-induced distress. Cancer diagnosis, particularly BTs, is a life-changing event that may induce high psychological distress in patients who receive it (130), with a negative impact on their QoL and overall survival (131–133). Distress symptoms experienced by patients with BT are various, manifesting as insomnia, fatigue, pain, loss of concentration (134), up to depression, anxiety, adjustment disorder, and post-traumatic stress disorder (16, 122, 135, 136). NCCN Practice Guidelines in oncology define distress as “a multifactorial unpleasant experience of a psychological (i.e., cognitive, behavioral, emotional), social, spiritual, and/or physical nature that may interfere with one’s ability to cope effectively with cancer, its physical symptoms, and its treatment. Distress extends along a continuum, ranging from common normal feelings of vulnerability, sadness, and fears to problems that can become disabling, such as depression, anxiety, panic, social isolation, and existential and spiritual crisis” (137). There is a high prevalence of psychological distress among patients with intracranial tumors, ranging from 5 to 27%. Its presence is associated with different factors, the certainty of tumor progression, poor disease prognosis, fear related to imminent death, and possible (long-lasting) functional limitations induced by BT (1, 131, 138–140). In this regard, Loughan and colleagues in a cross-sectional study on 105 PBT patients observed the presence of a high level of death anxiety in the majority of patients, which contributed significantly to their overall distress (139). Due to its implication with patients’ psychological well-being, the routine screening of distress in this patient population could be useful and may assist physicians in providing proper interventions (140–142) such providing emotional and psychological support to patients (3).

### 3.2 Impact of antiseizure medications

Another major aspect of the onset of neuropsychiatric symptoms in patients with BTRE could be due to ASM-related AEs (1, 16, 143). Behavioral side effects induced by steroids and ASMs occur in up to 10% of patients started on ASMs (110).

Unfortunately, data on first-generation ASM-induced behavioral AEs in BTRE patients are scarce (1, 35) and report phenobarbital-induced cognitive slowing (1, 34, 111), sedation for carbamazepine (1, 111), cognitive slowing and depression for phenitoyn (1, 112) fatigue for valproic acid (1, 111, 144–146) and tiredness for tiagabine (147) (Table 1).

Regarding second and third generation ASM-induced AEs in BTRE patients, data are more consistent. Psychiatric side effects seem to be particularly uncommon with lamotrigine (148), gabapentin (149), oxcarbazepine (111, 150), and vigabatrin (110), while levetiracetam and perampanel have been associated with aggressive behavior and anger (113, 151–167). Levetiracetam, which seems to be the preferred first-line choice in the treatment of BTRE (1, 8, 115, 168), can induce a higher rate of neuropsychiatric AEs such as aggressiveness, agitation, anxiety, and depression, compared to other ASMs (8, 17, 113, 115, 151–157, 166). Particularly, Bedetti and colleagues, in an observational, prospective, multicenter study on 259 BTRE patients, highlight that localization in the frontal lobe and treatment with levetiracetam is associated with a higher risk of neuropsychiatric AEs in this patient population (113); on the other hand, the association with valproic acid can mitigate AEs of levetiracetam on mood in BTRE patients (144, 145). Perampanel as add-on therapy proved to be effective in prospective and retrospective studies on BTRE patients, despite a moderate incidence of neuropsychiatric and behavioral AEs including aggressiveness, agitation, fatigue, and tiredness (158–165, 167). Topiramate can cause higher-than-expected incidence of psychiatric adverse events, including psychosis and aggressive behavior (110, 169–171). Only one study on brivaracetam seems available from scientific literature, where brivaracetam as add-on therapy showed moderate incidence (21.2%) of neuropsychiatric AEs (anxiety, agitation, fatigue), in a multicenter retrospective study on 33 BTRE (172). Good efficacy and tolerability were observed for lacosamide as mono or add-on therapy in BTRE patients, although fatigue, somnolence, confusion, and dizziness are reported (148, 173–180).

Other studies showed mood-modulating effects of ASMs. Oxcarbazepine in monotherapy showed good efficacy in controlling seizures and improving mood in patients with BTRE (150). Two studies on pregabalin in BTRE patients evidenced scarce incidence of neuropsychiatric AEs (151, 181). Actually, in an open before-after pilot study on 25 BTRE patients treated with pregabalin as an add-on, Maschio and colleagues observed an improvement in anxiety and QoL questionnaires’ scores (182). Scarce incidence of neuropsychiatric AEs is reported for zonisamide (183, 184) and lamotrigine (1, 148) (Table 1). Depression is most frequently associated with starting phenobarbital, vigabatrin, levetiracetam, felbamate, or topiramate, but can also be associated with discontinuation of ASMs such as carbamazepine, oxcarbazepine, valproic acid, and lamotrigine (110).

Data regarding the efficacy and tolerability of other ASMs as add-on therapy in BTRE is scarce: clobazam seems to have good efficacy with few AEs (185). Eslicarbazepine in non-oncological patients with epilepsy seems to improve mood and insomnia, ameliorating the patients’ perceived QoL (186), and preliminary data on BTRE patients suggest good efficacy with no behavioral AEs when used as an add-on (187). Only one, quite old study (149) evidenced the appearance of drowsiness in 1 out of 14 BTRE patients treated with gabapentin as add-on. No studies on cenobamate-related neuropsychiatric AEs are available in BTRE patient populations.

This evidence highlights how adequate management of ASMs therapy in BTRE patients should be focused on allowing maximum effectiveness with the lowest incidence of AEs in order to maintain a good QoL (1, 3). This implies not only monitoring medical aspects linked to the disease, but by considering and trying to preserve as much as possible all those apparently secondary factors such as the physical, cognitive, and behavioral sphere, which for the patient represent the center of his existence, of his sense of identity and of autonomy (1, 3). To achieve this goal, it is essential to optimize therapeutic choices in relation to individual characteristics and in collaboration between different specialists (3).

## 4 Quality of life issues

QoL is a complex and multidimensional concept that includes the integration and good functioning of different aspects of an individual’s life: physical, psychological, cognitive, social, and sexual, as well as the ability to perform daily life activities (3, 188–191). The World Health Organization defines it as: “the subjective perception of one’s position in life, in context of culture and values where you live, and in relation to own objectives, hopes, habits, and matters” (191). This concept has been well studied over the years especially in populations affected by chronic pathologies, due to the permanent impact that illness, together with pharmacological treatments, can have on patients’ global functioning.

### 4.1 Impact of brain tumor-related epilepsy and its treatment

BT and its treatment-related AEs can potentially alter the patients’ autonomy, mental abilities, and emotional state (1–3, 192, 193), with detrimental effects on self-perceived QoL. Regarding epilepsy, several factors beyond the abovementioned psychological and organic issues related to BT, BTRE and their treatments must be taken into account. Epilepsy adds a further burden to BTs, inducing even more disabling effects; indeed, epilepsy is considered the most important risk factor for long-term disability (1–3, 35). Studies on patients with non-oncological epilepsy report that epilepsy alters the patients’ perceived QoL through three main factors: (a) lack of autonomy caused by the temporary loss of control over one’s body and the surrounding environment, (b) possible social stigma and marginalization, and (c) AEs of ASMs. These three factors become even heavier to bear in patients who must confront both epilepsy and BT (1–3).

Experiencing an epileptic seizure is already a highly destabilizing factor for the patient with BT, as it represents not only a loss of autonomy and control over one’s body (as for all patients with epilepsy) but is also a constant reminder of the “tumor disease,” making the patient feel “different” and possibly leading to isolation and psychological complaints (1, 3). Moreover, a diagnosis of epilepsy not only implies that patients have to deal with the physical impact and unpredictability of seizures, but also have to cope with the associated and often negative social stigma (194), which can further exacerbate their actual or perceived marginalization. In fact, literature data highlights how epileptic seizures in patients with BT, especially when uncontrolled, have a negative effect on social and economic participation, morbidity, health-related QoL, neurocognitive functioning, and can result in patients’ loss of autonomy (e.g., driving license withdrawal) as well as higher distress for caregivers (143, 195). These aspects can therefore induce strong emotional distress and profound psychological suffering (3).

We have already mentioned how ASMs, the need for their long-term intake, the possible dangerous interactions with systemic therapies, and related AEs affect the patient’s physical, neuropsychological, and behavioral domain (1–3). Indeed, studies on patients with epilepsy (with or without BT) showed that the use of ASMs can worsen the perceived QoL (2, 3, 6, 31). Our study (6) evidenced that QoL was significantly influenced by the presence, type, and duration of ASM therapy, but not by the number of seizures in patients with BTRE; that means that whether experiencing one seizure or many, the patient’s perception of QoL did not change; what weighs on the patient is the diagnosis of epilepsy, which is accompanied by the other issues (6). First, patients assuming ASMs (independently of the ASM used) perceive significantly more negative effects on cognition, social function, and AEs of ASMs, with respect to patients who had not taken ASMs, and just taking ASMs results in significantly higher distress levels, regardless of other factors. This could mean that patients consider the antiepileptic therapy as a negative influence on their cognitive functioning and QoL, independently of the ASM used. Moreover, in this study, patients assuming ASM polytherapy perceive significantly more AEs of ASMs and have a worse perception of their health with respect to patients in ASM monotherapy. The duration of ASM therapy can have a significant influence on patients’ QoL as well; the longer the therapy, the more negatively the ASMs impacted patients’ social and cognitive spheres (6). These results are in line with those by Klein and colleagues who reported that patients with BTRE showed a worse QoL compared to BTs without epilepsy, due to the ASM-related AEs (34). The development of newer ASMs offer good efficacy with lower incidence of AEs resulting in increase or stability in patients’ perceived QoL test scores compared to the older ASMs (150, 164, 165, 182, 184, 196).

Considering the downfalls of ASMs and the efficacy of antitumor treatment in reducing seizures, ASM withdrawal after an interval of seizure freedom might be considered (31, 143). A prospective study in glioma patients showed that only 26% (12/46) of patients who were ≥ 1year seizure free from the date of last antitumor treatment, had a recurrent seizure after ASM withdrawal, compared to 8% (2/25) of patients continuing ASM therapy (median follow-up ∼2years) (197). In a retrospective ASM withdrawal study after tumor resection, 19% (3/16) of BTRE patients had a recurrent seizure (median follow-up ∼3years) (198). Italian BTRE guidelines suggest to withdraw ASM in case of prophylactic anti-seizure treatment, to not withdraw not in case of >2 year seizure freedom and BT progression or recurrence, and advise for future studies in case of >2 year seizure freedom and stable BT (31). Thus, ASM withdrawal can sometimes be considered in patients with BTRE, but optimal timing is currently unknown and potential benefits need to be weighted carefully against the potential risk of seizure recurrence, preferably in a shared decision-making process (31, 143, 199).

### 4.2 Sexual functioning

Sexuality is an important QoL domain; yet, it is rarely addressed by oncologists even though cancer and its treatment frequently affect sexual functioning and intimacy with a detrimental effect on patients’ feelings of desirability and self-perceived QoL (3, 190, 200–202). Some evidence pointed out that the incidence of sexual disturbances among cancer patients ranges from 40 to 100%; incidence rates vary greatly, depending on whether this problem is taken into account or not when caring for the patient (3). Because brain tumors do not affect sexual organs, physicians might not expect to observe sexual dysfunction, nor would they assess for it. However, all cancer therapies (surgery, CT, RT, hormonal therapies, and possibly immunotherapy) have the potential to significantly impair sexual functioning, an outcome which often remains distressing and unresolved even in survivors (200–202). Surbeck and colleagues evaluated sexuality in 32 BT patients who underwent surgical intervention and found that almost 50% suffer from sexual disturbances (203). It is therefore clear that sexuality is a problem that deserves attention due to its implication on patients’ psychological well-being. Common sexual disturbance usually includes achieving and sustaining orgasm: loss of sensation, erectile dysfunction for men, and pain during intercourse for women (3, 200, 201). Sexual problems can occur at any point during disease course: at diagnosis, during treatment, or during post-treatment follow-up, and are concerns for patients at all stages of disease progression (3, 200, 201, 204, 205). Unlike many other cancer treatment-induced AEs, sexual disturbances generally do not resolve in the first 2 years of disease-free survival but can remain constant and relatively severe (3, 189, 200, 201).

Evidence on sexual disturbances in BTRE is scarce, consequently, their incidence could be underestimated; nonetheless maintaining a satisfactory sexual relationship is a fundamental aspect of a good QoL (3, 189). In BTRE patients, sexual disturbance can occur as ASMs-related AEs. For this reason, the possible effects of ASMs on the sexual sphere must be considered by clinicians, and the choice of the drug should be made monitoring this aspect (3, 206).

Regarding epilepsy, sexual disfunction seem particularly common yet under-discussed by healthcare professionals (207, 208). Many symptoms are related to ASM’s AEs, including hypersexuality, hyposexuality, ejaculatory dysfunction, and erectile dysfunction. Enzyme-inducing ASMs and valproic acid may produce high incidences of decreased libido; topiramate, pregabalin and gabapentin may cause sexual disfunctions, whereas oxcarbazepine, lamotrigine and levetiracetam may improve sexual function, but data are preliminary (208). In a study evaluating the incidence and clinical associations of sexual dysfunction in 89 adult epilepsy patients, self-identifying as overweight/obese or taking strong enzyme-inducing ASMs were the only two independent factors predicting sexual dysfunction (207).

Regarding BTRE, there are no studies on the effect of ASMs on the sexual sphere in these patients to date. The only study available is a case report on reversible zonisamide add-on-induced erectile dysfunction in a patient with oligoastrocytoma, in which the authors observed a total remission after drug withdrawal (206). Further studies are necessary to better understand and treat BT-, epilepsy-, and treatment-related sexual dysfunction in patients with BTRE.

Regarding the evaluation of sexual dysfunctions in BT and BTRE, there are no BT- and BTRE-specific assessment tools available, although the development of specific measures could be useful in addressing these issues (209). Different instruments have been used in the few studies available; it is important to evaluate these aspects in patients with BTRE even using non-specific tools, but the results must be considered cautiously. Identification and treatment of sexual problems are important issues for patients because sexual dysfunction may alter relationship intimacy, increase emotional distress, lead to a negative body image, or be perceived as a constant reminder of one’s cancer history, and detecting the presence and severity of sexual concerns should be considered part of treatment and follow-up care (3, 190, 200, 201, 205, 206, 209). This should encourage clinicians to realize that BTRE patients, in addition to the disease, have intimate and personal issues which are affected by the illness and by each treatment, and, for this reason, these aspects deserve to be evaluated to better take care of the patient in their globality.

## 5 Tools and methodology

Neuropsychological assessment of BTRE patients must integrate the knowledge of tumor and epilepsy and it must be able to detect and quantify the impact of both illnesses and their treatments on the cognitive, emotional, behavioral, and QoL of the patient (31). The goal of neuropsychological evaluation is to obtain as much information as possible about a patient’s functioning at a specific time to both monitor cognitive profile and refer patients to proper neuropsychological rehabilitation programs in case of deficits (3, 13, 31, 36, 43).

### 5.1 Domains and timing of assessments in brain tumors

Giovagnoli et al. identified the main cognitive domains and the proper neuropsychological tests sensitive to BT damage and treatment (43). According to the author, neuropsychological evaluation has to explore the following domains: attention (selective, divided, interference control), visuo-motor coordination and motor speed, executive functions (set-shifting and flexibility, abstract reasoning), and short- and long-term verbal- and visuo-spatial learning (43). Concerning the memory domain, Durand and colleagues observed in a retrospective study on 158 BT patients that almost 92% of patients showed impairment in episodic memory, particularly in retrieval processes, while deficits in storage and encoding processes were less prevalent (210). According to the authors, this pattern was similar across patients with BT regardless of tumor histology and treatment modalities. For this reason, the authors suggested that the assessment of all three components of episodic memory should be part of the regular neuropsychological evaluation in the patient population (210).

**Figure 1:**
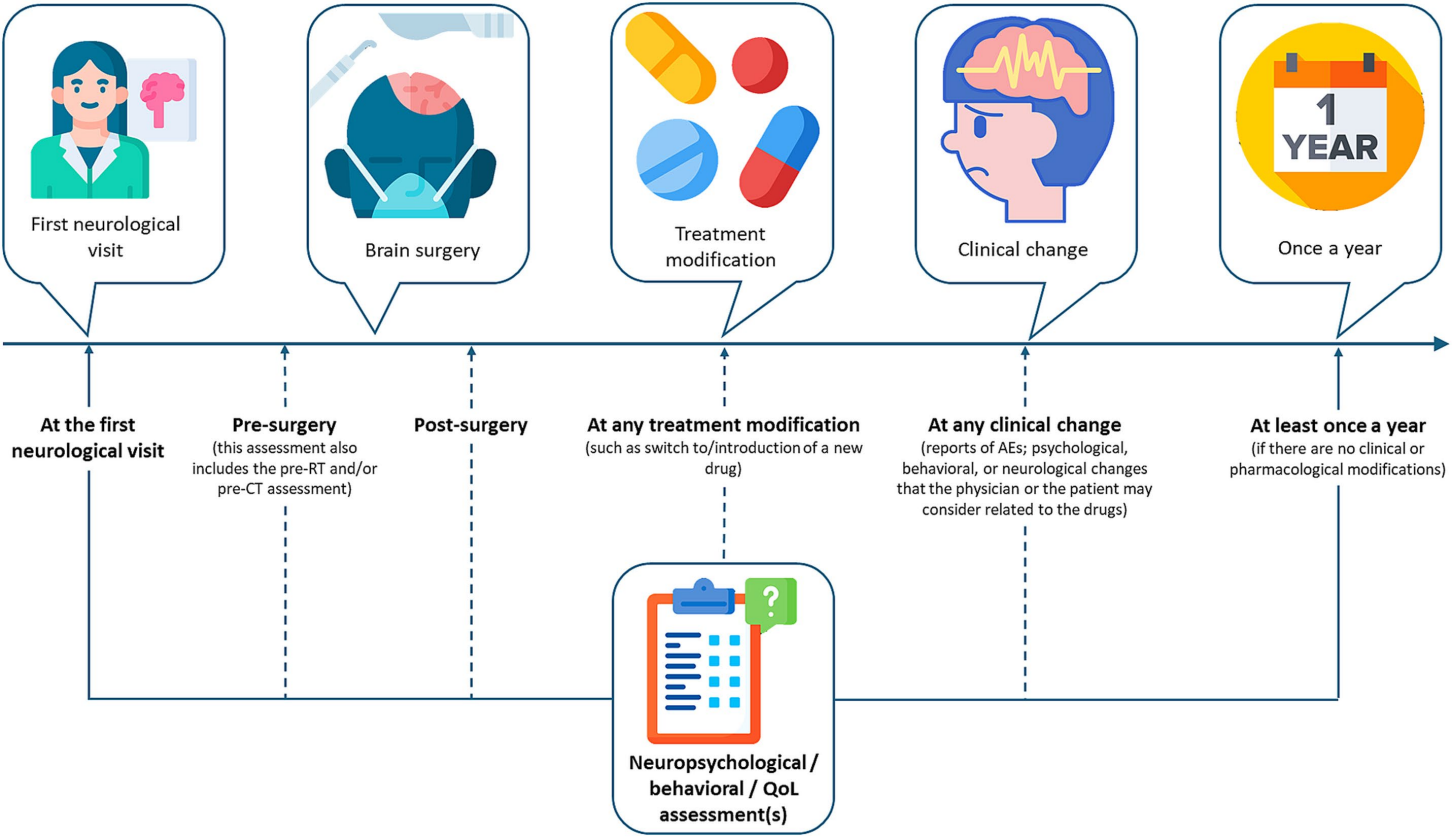
Possible timings of neuropsychological, behavioral, or QoL assessments. Patients with BTRE should undergo a complete evaluation at the first neurological visit and once a year; this will allow to understand the patient’s improvement overtime beyond tumor progression and seizure control, without stressing the patient with too many evaluations. Further assessments can be carried out pre-/post-surgery, when treatment is modified, or at any clinical changes, if the patient, caregiver, and/or treating physician suspect deterioration. These assessments should investigate the specific neuropsychological, behavioral, or QoL domain suspected of being impaired. Any assessment deemed necessary at treatment modification should be coordinated and agreed upon with other specialists (neurologist, epileptologist, oncologist, radiologist, etc.) to avoid repetition of the same test over short periods of time.

The timing of neuropsychological evaluation can be different according to clinical objectives. Literature data highlight that neuropsychological assessment in BT patients should be administered at the time of diagnosis and before and after any treatments (43, 93). However, this approach would result in a sequela of assessments (e.g., at diagnosis, at the start and the end of CT, RT, and surgery, before and after an ASM switch, etc.), possibly overstressing the patient and repeating the same tests over time (see Figure 1). As such, it would be important to identify common evaluation timepoints among each treatment. Monitoring neuropsychological status through patients’ reported outcomes at each follow-up visit could allow physicians to refer patients to neuropsychological testing only if the occurrence of impairment is suspected/reported and not before and after any treatment.

#### 5.1.1 Evaluation at diagnosis

At BT diagnosis, a neuropsychological evaluation is a helpful tool instrument to detect the possible effect of BT in patients with otherwise normal neurological status; provide detailed information on patients’ neuropsychological and behavioral functioning; orient clinical decision-making to choose adequate surgery procedures; monitor post-surgical changes and treatments’ AEs and compare them to the baseline evaluation; and implement neuropsychological rehabilitation programs in case of neuropsychological deficits (3, 13, 36).

In patients with BT who are eligible to undergo total or partial tumor resection, different evaluation steps are identified: pre-surgical, post-surgical, and long-term follow-up, as well as intraoperative monitoring in the case of awake surgery (36).

#### 5.1.2 Presurgical evaluation

Pre-surgical neuropsychological assessment should include a battery of tests evaluating both right and left hemisphere functions (32). This includes an accurate screening of all language domains (naming, comprehension, repeating, reading, writing, phonological discrimination, verbal fluency, apraxia), processing speed, verbal short-term memory span, non-verbal abstract reasoning, visuo-spatial short-term memory span, visual selective attention, set-shifting, visuo-spatial abilities, visuo-constructive skills (32).

#### 5.1.3 Intraoperative monitoring

After establishing the patient’s pre-surgical, baseline cognitive performances, intraoperative monitoring is required in the case of awake surgery. Generally, few neuropsychological brief tests covering cognitive domains corresponding to the site of the surgical intervention and suitable to be administered intraoperatively are selected. A baseline of these tests is obtained the day before surgery and during the intervention itself. The choice of tests is focused on covering the domains of orientation, comprehension, object/action naming, verbal fluency, and other specific cognitive areas (37). In a systematic review by Ruis (211), it is reported that almost 90% of the studies examined were focused on the assessment of language functions at this stage. Tests for visuospatial functions, learning and memory, calculation, and emotions are also reported, but in just a few studies; specific tasks for the evaluation of other cognitive domains such as face recognition, executive functions, musical skills, and finger gnosis are rarely used (211).

#### 5.1.4 Postoperative evaluation

Postoperative evaluation administered in the immediate post-surgery period is aimed at monitoring recovery (3), documenting the extent of cognitive changes between pre- and post-surgery (37), and identifying residual deficits that may benefit from a rehabilitation treatment (3, 43). The timing of post-operative monitoring may change depending on the medical condition and clinical changes observed by the clinician or reported by the patient. It may occur immediately after surgery if inpatient rehabilitation is necessary, but a period of few days to 2–3 weeks of postsurgical recovery is usually advised (36).

#### 5.1.5 Long-term evaluation

Long-term monitoring of neuropsychological profiles with serial evaluations at 6-month, regular intervals is helpful as part of comprehensive brain tumor management (3). As cognitive impairments, more than other neurologic symptoms, can have a significant impact on a patient’s perceived QoL, periodic monitoring can help mitigate the negative impacts of cognitive and behavioral disturbances on QoL (36) by guiding therapeutic choices (pharmacological or non-pharmacological) (3, 43, 88). Tests for attention, executive functions, and memory can detect the main BT-related cognitive deficit, and, among these, some have clinical and prognostic significance (43). Similarly, Taphoorne and Klein (7) indicate in their hierarchic model that tests for gliomas must assess perception, information processing, attention, executive, memory, and intellectual abilities.

### 5.2 Assessment tools in brain tumor-related epilepsy

Tables 3–6 report all tests mentioned in this section, alongside the related standardization and validation studies.

**Table 3:** Neuropsychological tests used in patients with BTRE and tests’ standardization and validation studies.

Neuropsychological function	Neuropsychological test	References
Global cognitive status	Mini mental state examination (MMSE) Montreal cognitive assessment-MOCA	(2, 13, 32, 33, 150, 184, 225, 260–263)
Non-verbal abstract reasoning	Raven colored progressive matrices	(2, 13, 32, 163, 264)
Attention (selective, set-shifting, switching, sustained attention)	Visual search Trail making test Stroop color-word test Letter digit substitution test Letter–digit modalities test Digit symbol substitution test	(2, 13, 34, 163, 184, 265–270)
Executive functions	Tower of London Fab-frontal assessment battery Concept shifting test Categoric word fluency task Phonetic and semantic fluency Controlled oral word association test	(2, 13, 32, 34, 163, 184, 265, 270–273)
Language	Battery for the analysis of aphasic deficits-BADA Boston naming test Token test	(2, 32, 271, 274–276)
Verbal and visuo-spatial learning and long-term memory	Short story Visual verbal learning Rey auditory verbal learning test Rey Osterreith complex figure Memory comparison	(2, 13, 32, 163, 184, 265, 266, 270, 273)
Verbal and visuo spatial short-term memory	Digit span (forward/backward) Corsi span (forward/backward) Working memory task	(13, 32, 34, 265, 268, 273, 277)
Visuo-spatial abilities	Rey Osterreith complex figure-copy Clock drawing test Constructive apraxia Line bisection test Facial recognition test Judgement of line orientation test Bit-behavioral inattention test	(2, 13, 34, 258, 265, 266, 273, 278, 279)

**Table 4:** Behavioral tests used in in patients with BTRE and tests’ standardization and validation studies.

Neuropsychiatric symptoms	Neuropsychiatric scales	References
Depression	HADS—Hospital anxiety and depression scale Zung self-depression rating scale Beck’s hopelessness scale BDI-II—Beck depression inventory	(150, 165, 173, 181, 182, 213–217, 225, 262, 263, 269, 280)
Anxiety	HADS—Hospital anxiety and depression scale HAM-A—Hamilton anxiety scale STAI—State-trait anxiety inventory	(182, 213, 215, 218, 280, 281)
Irritability	Aggression questionnaire	(165, 219)
Global symptoms scales	Symptom check-list 90 Neuropsychiatric inventory	(2, 113, 163, 220, 221)
Quantification of ASMS’ AEs	AEP—Adverse event profile	(2, 33, 147, 150, 163, 169–171, 182–184, 280)

**Table 5:** QoL test used in in patients with BTRE and tests’ standardization and validation studies.

QoL and clinical condition	QoL assessment tools	References
QoL in cancer	EORTC QLQ C-30 Functional Assessment of Cancer Therapy (FACT-G and FACT-BR) Short Form 36-SF 36 EURO QOL 5D	(33, 34, 150, 173, 182, 184, 225, 231, 262, 269, 273, 282–287)
QoL in brain tumors	EORTC BN-20	(231, 262, 273, 286)
QoL in epilepsy	QOLIE-31-P	(2, 33, 150, 163, 165, 173, 182, 184, 225)

**Table 6:** Sexuality questionnaires useful in BTRE patients and tests’ standardization and validation studies.

Sexual functioning assessment tools	References
FEMALE Sexual Function Index (FSFI) International Index for Erectile Function (IIEF) Premature Ejaculation Profile (PEQ) Peyronie’s Disease Questionnaire (PDQ) The Arizona Sexual Experience Scale (ASEX)	(231–237)

#### 5.2.1 Neuropsychological assessment tools

Regarding the cognitive sphere, particular attention should be given to the assessment of domains that are most affected in BT and epilepsy: memory, executive functions, and language. (3). More recent evidence included in neuropsychological evaluation the assessment of global cognitive status, attention and information processing speed, planning and problem-solving abilities, abstract reasoning, and visuospatial abilities (2, 13, 156), and social cognition (212). Several neuropsychological assessment tools are reported in Table 3.

#### 5.2.2 Emotional and behavioral assessment tools

Regarding the evaluation of emotional and behavioral issues in BTRE patients, studies have usually used self-administered, disorder-specific questionnaires (150, 165, 173, 181, 193, 213), and only a few studies report the use of global symptom scales (2, 113, 163). Disorder-specific tools such as the Zung self-depression rating scale (214), the Hospital Anxiety and Depression Scale (HADS) (215), Beck’s hopelessness scale (BHS) (216), Beck’s Depression Inventory (BDI) (217), State–Trait Anxiety Inventory-STAI (218), Aggression Questionnaire (219) have the advantage of deeply exploring a specific psychopathological dimension. Global symptom scales, on the other hand, could highlight a wide range of behavioral and emotional symptoms experienced by patients, although the results must be interpreted adequately and integrated with an interview, in both cases. Among global neuropsychiatric symptom scales, the Neuropsychiatric Inventory (220) and Symptom checklist 90 (221) have been used (2, 113, 163). The Neuropsychiatric Inventory is used to evaluate the psycho-behavioral disorders associated with cognitive deterioration, and to evaluate the stress load to which the patient subjects family members, caregivers and professional staff; although originally created for dementias, it is one of the most used scales for behavioral disorders in neurology (220).

Several emotional and behavioral assessment tools are reported in Table 4.

#### 5.2.3 Assessing treatment-related adverse events

Serial neuropsychological evaluations can be able to detect cognitive AEs related to all treatments, which can be an early indicator of tumor recurrence (even prior to radiographic progression) (3, 7, 43, 88, 222). The neuropsychological evaluation in BTRE patients is fundamental, especially related to ASMs which can induce cognitive, emotional, and behavioral AEs impairing patients’ QoL more than seizure frequency, a main concern during the survival from BT (3, 34, 223). Patients with BTRE showed in fact lower cognitive performance in psycho-motor function, information processing speed, working memory, and executive functions compared to patients with BT not taking ASMs (3, 34). AEs are higher in patients undergoing polytherapy, treated with old-generation ASMs, or taking ASMs long-term, while they are lower in patients undergoing monotherapy and/or with new-generation ASMs (33). Neuropsychological and neuropsychiatric evaluation can provide useful information for “epileptological decision making” to choose the most appropriate drug and monitor ASMs’ possible AEs in order to improve patients’ QoL (31). Consequently, neuropsychological evaluation should be administered before ASM introduction and at any ASM modification due to inefficacy, AEs, or drug-to-drug interactions (31), as well as periodically during ASM treatment.

The identification and evaluation of the possible ASMs AEs, both in BTRE and non-oncological epileptic patients, a rapid and useful tool is the Adverse Event Profile (224). This self-report questionnaire is aimed to evaluate the frequency and intensity of possible AEs experienced by patients through a Likert scale (1–4 points). The final score (range: 19–76) indicates the total AEs’ burden of ASM therapy; higher scores indicate more severe AEs (3, 224) (see Table 4).

#### 5.2.4 Quality of life assessment tools

QoL reflects a patient’s personal perception of different functioning domains, and, for this reason, it needs to be assessed through “self-report” tools (224). Several instruments have been developed over the years; however, to date, there is no questionnaire or scale specific for BTRE patients (3). All available tools investigate patients’ perceived QoL taking into account BT and epilepsy separately, without considering their combined effect, as it occurs in BTRE patients. Nonetheless, recent evidence indicated how the QOLIE 31-P, specific for patients with epilepsy, can represent an adequate alternative tool to assess QoL in those with BTRE as well (2, 3, 33, 150, 163, 165, 173, 183, 196, 225). QOLIE 31-P contains seven multi-item scales that tap the following health concepts: emotional well-being, social functioning, energy/fatigue, cognitive functioning, seizure worry, medication effects, and overall QoL. The QOLIE-31 also includes a single item that assesses overall health. A QOLIE-31 overall score is obtained using a weighted average of the multi-item scale scores. Higher scores reflect a better QoL; lower ones, a worse QoL (226).

On the other hand, the European Organisation for Research and Treatment of Cancer (EORTC) QoL group developed two tools to assess QoL in cancer patients. The first one is the EORTC QLQ-C30 (227) a 30-item measure, designed to assess the health-related QoL of patients with cancer. The second one is the EORTC QLQ-BN20, specifically developed and validated for patients with brain cancer, which includes 20 items assessing visual disorder, motor dysfunction, various disease symptoms, treatment toxicity, and future uncertainty (228). This tool, in combination with the EORTC QLQ-C30, is often used in clinical trials in glioma patients undergoing CT and radiation therapy. The items on both the EORTC QLQ-C30 and the EORTC QLQ-BN20 measures are scaled, scored, and transformed to a linear scale (0–100). Differences ≥10 points in a health-related QoL parameter are classified as clinically meaningful changes. Changes >20 points are classed as large effects.

Another tool used to evaluate patients’ health-related QoL is the Functional Assessment of Cancer Therapy-Brain (FACT-Br) a subscale of the FACT-General (FACT-G) questionnaire. The FACT-G was developed to provide information about health status that is specific to patients with cancer (229). FACT-Br was developed as a new combined brain subscale questionnaire (230). FACT-Br subscale, the brain tumor-specific version, is a 23-item questionnaire that can be completed in 5 to 10 min with little or no assistance by patients who are not neurologically incapacitated. This brain subscale is usually used along with the core (general) questionnaire that includes 27 items (229, 230). Patients rate all 5 items using a five-point Likert scale ranging from 0 “not at all” to 4 “very much.” Overall, higher ratings suggest higher QoL. Items are totaled to produce the following subscales, along with an overall QoL score: physical well-being (7 items); social/family well-being (7 items); emotional well-being (6 items); functional well-being (7 items); and concerns relevant to patients with BT (23 items).

Several QoL assessment tools are reported in Table 5.

#### 5.2.5 Sexuality assessment tools

As mentioned above, the assessment of sexuality is not included in the standard care of patients with BTRE, and no specific tool is available. Literature data indicate that the evaluation of this domain deserves attention during the entire disease trajectory of this particular patients’ population (3, 190, 206, 209). Studies on this topic used sexual sphere tools developed on non-oncological populations which are more focused on the physical dimension, rather than the psychological dimension, and do not explore aspects such as the possible alteration of patients’ body and self-image, an aspect that is fundamental in BTRE patients. Nonetheless, the use of sexual domain assessment tools, although not specific, allows obtaining quantitative data regarding this domain.

To date, the tools used in BT patients are few (See Table 5) (231–237); among the, we believe that the most useful could be the Female sexual function index (FSFI) for women and the International Index of Erectile Function (IIEF) for men (232, 233). Both are simple, short, rapidly administrable and assess not only a single sexual symptom, but provide an overall score of sexual global satisfaction. FSFI includes 5 subscales: desire, arousal, lubrication, orgasm satisfaction, and total sexual functioning; IIEF is also composed by 5 subscales, namely erectile dysfunction, orgasm, desire, intercourse satisfaction, and overall satisfaction.

Despite the usefulness of these scales, we further stress the need for studies on this topic in BTRE as there are no scales or questionnaires for the assessment of sexual sphere in this specific patient’s population.

Several sexuality assessment tools are reported in Table 6.

## 6 Future directions

The recent, numerous advances made in the field of neuroscience have prompted the expansion of brain functioning knowledge in normal and pathological conditions, including tumoral epilepsy. If, on one hand, this allowed physicians to better understand the etiopathogenesis of the disease, on the other it drew attention to the numerous difficulties that these patients experience in their daily life, as disease-related consequences. This should lead healthcare professionals to no longer consider the single aspects of illness as separate entities (BT, systemic therapies, epilepsy, ASMs), but to consider the disease as a whole. A patient with BTRE has a specific and multimodal disability due to the simultaneous presence of different factors that cannot be separated, and their concurrent association constitutes the “BTRE-induced disability.” We believe that considering this aspect can represent a basis for better care of BTRE patients, with specialists from different areas addressing all aspects of the patient’s life (31).

In this perspective, we believe that patients with BTRE would benefit from a shift of perspective on their condition, and that BT, epilepsy, and especially BTRE should be integrated into a biopsychosocial model rather than just a biomedical model. While the latter might focus on the illness’s biological factors, seizure and tumor freedom, and medical interventions, the former incorporates psychological and social factors to the biological ones, and suggests how the individual’s experiences and expectations can influence health and illness (238–240). This model emphasizes the dynamic interactions between neuro-cognitive factors, psychological processes, and the social environment, and have been usefully applied to other chronic health conditions, including brain injury (241), (brain) cancer (242, 243), Alzheimer’s disease (244) and especially chronic pain (245, 246).

The complex neurological, psychological, and social downfalls of BTRE highlight the importance of adopting such standpoint to understanding factors impacting the patients’ QoL, and thus offers a framework to address the illness in a more comprehensive and multimodal approach which takes into accounts the patients’ needs. To our best knowledge, there seems to be no studies framing BTRE within the biopsychosocial model to date, but some are available for epilepsy or BT alone (242, 247). In people with epilepsy, growing evidence suggests that psychosocial factors and poor mental health – and not clinical variables such as age at onset, seizure frequency, and AEs from ASMs – have the greatest impact on quality of life; moreover, these patients tend to view their handicaps as psychological rather than purely physical and complain about a lack of counseling and support (247). Indeed, the biopsychosocial model explains a significantly larger amount of variance in QoL compared with the biomedical model alone and, within the biopsychosocial model itself, the psychological and social domains still explained a greater amount of the variance in QoL compared with the biomedical model (247).

Recent evidence showed how cognitive rehabilitation with the goal to improve autonomy, self-awareness, emotional coping strategies, and management of cognitive impairments in BTRE patients may have an important role in achieving both an improvement in neurocognitive and behavioral functions and a better QoL after treatment, ultimately enhancing the social and professional integration of patients (2, 13, 248–251). Some supportive psychological interventions for patients with BT with and without epilepsy have been implemented in the last decades with the aim of maintaining good QoL and psychological well-being, showing how different types of therapeutical approaches could be useful in the treatment of anxiety, depression, distress, and social isolation (2, 252); such interventions comprise psycho-social interventions, mindfulness training, individual support, supportive meeting groups, and problem-solving strategies training (252, 253). Some studies also highlight how integrated interventions can be useful in taking care of these patients. In a prospective pilot study on BTRE, implementing a pathway that included epileptological visits, neuropsychological rehabilitation, patients’ psychologically supportive meeting groups, and social assistance can induce a significant improvement in patients’ QoL (2). Unfortunately, this type of approach requires considerable resources, specialized personnel, and a high level of compliance and commitment from patients and their caregivers. A solution could be represented by remote therapeutic interventions. Recent advances in computer technologies and the COVID pandemic have favored the introduction of telerehabilitation as a new treatment methodology that allows to provide remote neuropsychological and/or emotional support to patients (254–256). Literature studies show how telerehabilitation is a feasible, valid, and effective tool that can allow patients easy access to services, guaranteeing continuity of care (254–256). In this regard, most seizures within BTRE are self-limited, and many can be managed safely at home. Focused educational intervention regarding home management of seizures may be effective in providing patients and caregivers with a sense of control over an unpredictable condition, reducing distress, and improving their awareness on seizure management, thus prompting a more aware use of acute care services and reducing the number of Emergency Department visits and hospital admissions. Patients’ and caregivers’ education can play an important role in improving outcomes, and this intervention should be incorporated in their routinely appointments with the medical staff (257). Finally, it is important to remember that within BTREs, prompt recovery is not possible: time is fundamental for both the treating physicians, to truly understand the patient’s needs and how treatment works, and for the patient, who has to adjust and learn how to cope with the new challenges BTRE brings.

## 7 Conclusion

BTRE-related neuropsychological and behavioral issues can be a direct consequence of BT, epilepsy, and their treatments such as ASMs, CT, RT, or corticosteroids, which can alter the structure of specific brain areas and/or networks. On top of this, emotional aspects following BTRE diagnosis, such as the possible loss of autonomy, poor prognosis, and fear of death, can induce behavioral and emotional disturbances. These factors can significantly alter patients’ self-perceived QoL (2) with an impact on overall survival (85–87, 103–105). For this reason, the main objective in taking care of these patients beyond improving survival is to maintain a good QoL throughout the disease trajectory, helping patients to resume their lives as much as possible (1–3, 5, 258). That implies the use of adequate psychometric batteries for the assessment of neuropsychological, emotional, and QoL issues, including sexuality, to help guide treatment and rehabilitation. Although there are no tests specifically designed for BTRE patients, those currently used in this patient population can still provide reliable indices of the patient’s functioning. This could be useful not only to monitor efficacy and/or treatment-related AEs, but also to provide a baseline for setting appropriate therapeutic support interventions in case of neuropsychological impairment or psychological suffering.

In Figure 1, we provide a flowchart suggesting the appropriate timing for neuropsychological, behavioral, and QoL assessment; in Table 2, we provided considerations to help clinicians choosing the appropriate ASMs taking into account their neuropsychiatric or neuropsychological AEs.

Our review is the first to comprehensively explore neuropsychological, behavioral, and QoL issues in patients with BTRE, as well as the possible assessment methodology. Although our findings offer some insights, further research is needed to establish causality and deepen our understanding of the interplay between all these variables and our intervention in terms of diagnosis, treatment, and timing of both.
